# Flat-arched feet display altered foot kinematics compared to normal-arched feet during walking

**DOI:** 10.1186/1757-1146-4-S1-P43

**Published:** 2011-05-20

**Authors:** Pazit Levinger, George S Murley, Christian J Barton, Matthew P Cotchett, Simone R McSweeney, Hylton B Menz

**Affiliations:** 1 Musculoskeletal Research Centre, Faculty of Health Sciences, La Trobe University, Bundoora, VIC, 3086, Australia; 2 Department of Podiatry, Faculty of Health Sciences, La Trobe University, Bundoora, VIC, 3086, Australia

## Background

Foot posture is thought to influence predisposition to overuse injuries of the lower limb. Although the mechanisms underlying this proposed relationship are unclear, it is thought that altered foot kinematics may play a role. Therefore, this study was designed to investigate differences in foot motion between people with normal and flat-arched feet using the Oxford Foot Model (OFM).

## Methods

Nineteen participants with normal- and flat-arched feet were recruited for this study (10 with normal and 9 with flat-arched feet). A foot screening protocol comprising measurements from weightbearing antero-posterior and lateral foot radiographs were used to classify foot posture. Tri-planar motion of the tibia, rearfoot and forefoot during barefoot walking were recorded from 10 cameras and evaluated using a three-dimensional motion analysis system incorporating a multi-segment foot model (OFM).

## Results

During midstance phase, participants with flat-arched feet demonstrated greater forefoot abduction (-12.9° ± 6.9° vs -1.8° ± 6.3°; p = 0.002), rearfoot internal rotation (10.6° ± 7.5° vs -0.2° ± 9.9°; p = 0.018) and a trend towards increased rearfoot eversion (-5.8° ± 4.4° vs -2.5° ± 2.6°; p = 0.06), compared to those with normal-arched feet. During late stance, participants with flat-arched feet demonstrated greater peak forefoot plantarflexion (-13.7° ± 5.6° vs -6.5° ± 3.7°; p = 0.004) and decreased peak forefoot adduction (-7.0° ± 9.2° vs 5.6° ± 7.3°; p = 0.004) compared to those with normal-arched feet.

## Conclusions

The findings of this study indicate that there are significant differences in sagittal and transverse plane movement of the forefoot and the transverse plane movement of the rearfoot between participants with normal- and flat-arched feet. These findings support the notion that those with flat-arched feet demonstrate altered motion associated with greater pronation during gait; factors that may increase the risk of overuse injury.

